# Intracavernosal UC-MSC secretome therapy for severe erectile dysfunction: a clinical anatomy pilot study with elastography and Doppler assessment

**DOI:** 10.3389/frph.2026.1775672

**Published:** 2026-04-21

**Authors:** Ria Margiana, Supardi Supardi, Maria Paulina Budyandini Dyah Pramesti, Heri Suroto, Reny I'tishom, Fatkhurrohmah Leo Amalia, M. Ikhsan Nugroho, Andi Ahmad Thoriq Pratama, Muhammad Hidayat Surya Atmaja, Kristophorus Jonathan Wibowo, Muhamad Rizqy Fadhillah, Tania Graciana, Wahyuning Ramelan, Ahmad Aulia Jusuf

**Affiliations:** 1 Department of Anatomy, Faculty of Medicine, Universitas Indonesia, Jakarta, Indonesia; 2 Andrology Education Program, Faculty of Medicine, Universitas Airlangga, Surabaya, Indonesia; 3 Dr. Soetomo General Academic Hospital, Surabaya, Indonesia; 4 Department of Orthopaedics, Faculty of Medicine, Universitas Airlangga, Surabaya, Indonesia; 5 Department of Biomedical Science, Faculty of Medicine, Universitas Airlangga, Surabaya, Indonesia; 6 Department of Radiology, Faculty of Medicine, Universitas Airlangga, Surabaya, Indonesia; 7 Medical Study Program, Faculty of Medicine, Universitas Airlangga, Surabaya, Indonesia; 8 Department of Pharmacology and Therapeutics, Faculty of Medicine, Universitas Indonesia, Jakarta, Indonesia; 9 Doctoral Program in Biomedical Science, Faculty of Medicine, Universitas Indonesia, Jakarta, Indonesia; 10 Department of Biology, Faculty of Medicine, Universitas Indonesia, Jakarta, Indonesia; 11 Department of Histology, Faculty of Medicine, Universitas Indonesia, Jakarta, Indonesia

**Keywords:** andrology, erectile dysfunction, mesenchymal stem cells, secretome, umbilical cord

## Abstract

**Introduction:**

Erectile dysfunction (ED) is often treated with phosphodiesterase type 5 inhibitors (PDE5i). However, some patients do not respond effectively to this treatment, making regenerative therapy a potential alternative. This study aims to evaluate the safety and efficacy of umbilical cord-derived mesenchymal stem cell (UC-MSC) secretome in patients with severe ED who are unresponsive to sildenafil therapy.

**Methods:**

In this clinical anatomy pilot study, 15 male patients with severe ED unresponsive to sildenafil were administered intracavernosal injections of 0.5 cc of UC-MSC secretome into each corpus cavernosum at the Andrology Clinic of RSUD Dr. Soetomo (Dr. Soetomo General Academic Hospital) in Surabaya, Indonesia, between January 2024 and May 2024 and at RS Universitas Indonesia between January 2025 and May 2025. Therapy safety was evaluated based on local bleeding, hematoma, infection, and pain, while efficacy was measured using the Erection Hardness Score (EHS), International Index of Erectile Function-5 (IIEF-5), reports of morning erections, stretched penis length (SPL), flaccid penis length (FPL), penis circumference, and Penile Doppler Color Ultrasound to evaluate peak systolic velocity (PSV), resistive index (RI), intima-media thickness (IMT), shear wave elastography (SWE), and strain ratio (SR).

**Results:**

Significant improvements were observed in EHS, IIEF–5, morning erections, SPL, FPL, penis circumference, PSV, SWE, and SR after therapy. Pain was temporary and subsided within a few minutes. No significant differences in local bleeding, hematoma, and infection were observed. There was a difference in the IMT of the cavernous artery that was not statistically significant, while RI could not be analyzed.

**Discussion:**

These findings suggest that UC-MSC secretome is safe and effective as an alternative therapy for severe ED unresponsive to sildenafil, as evidenced by significant changes in EHS, IIEF-5, morning erections, SPL, FPL, penis circumference, PSV, SWE, and SR post- therapy with the potential for temporary pain. Further research is needed to support the development of regenerative therapy for the treatment of ED unresponsive to conventional treatments.

## 1 Introduction

Erectile dysfunction (ED) is a medical condition characterized by the inability to achieve or maintain an erection sufficient for satisfactory sexual activity (1, 2). To meet the diagnostic criteria for ED, symptoms must persist for at least 3 months (2–4). In Indonesia, the prevalence of ED continues to rise, particularly among men over the age of 40, largely because of unhealthy lifestyles, psychological stress, and chronic systemic diseases such as diabetes and hypertension. Despite this increasing burden, social stigma often prevents patients from seeking treatment, underscoring the importance of health education and access to appropriate medical intervention (5).

Globally, the rate of prevalence of ED reaches 19.2%, with a marked increase in older age groups. Large epidemiological studies have reported that 41.5% of men experience ED, and 7.5% suffer from its severe forms (6, 7). In Indonesia, the prevalence rate among men aged 20–80 years is 35.6%, spanning mild to severe levels (6). These data emphasize the urgency of improving management strategies, especially for patients with severe ED who show inadequate response to sildenafil and other standard phosphodiesterase type-5 inhibitors (PDE5i) (7, 8).

From a clinical anatomy perspective, severe ED is often associated with structural and functional alterations in the corpus cavernosum, cavernosal smooth muscle, endothelial function, and neural integrity that impair the nitric oxide–cGMP pathway. When anatomical and vascular damage progresses beyond pharmacologic reversibility, PDE5i-based treatment, such as sildenafil, frequently fails to restore erectile function (9). Patients with severe organic ED and advanced structural and vascular damage may find PDE5i and conventional intracavernosal pharmacotherapy insufficient because of irreversible tissue remodeling. In this context, regenerative-based therapies have emerged as a promising approach to target the underlying endothelial, smooth muscle, and neural injury rather than providing only symptomatic vasodilation. The secretome derived from umbilical cord mesenchymal stem cells (UC-MSC) contains bioactive molecules with angiogenic and neurotrophic properties, including vascular endothelial growth factor (VEGF), brain-derived neurotrophic factor, and nerve growth factor, which have the potential to restore penile vascular perfusion and nerve function (10). Preclinical studies have documented the anti-inflammatory profile and safety of the UC-MSC secretome and demonstrated its ability to improve erectile function through vascular and neural remodeling in animal models (11, 12).

Safety and efficacy evaluation in patients with severe ED is essential to prevent complications such as local bleeding, hematoma, infection, and procedure-related pain (13). Erectile function was assessed using clinical scoring tools—Erection Hardness Score (EHS), International Index of Erectile Function-5 (IIEF-5), and morning erections—to provide a comprehensive functional overview (14, 15). Anatomical and physical parameters were also evaluated to reflect soft tissue and corporal body changes relevant to sexual function. In addition, penile Doppler Ultrasound was used to directly represent penile arterial perfusion and vascular wall condition (16). Integrating functional, anatomical, and vascular measurements enables differentiation between organic and psychogenic etiologies of ED, while also determining the therapeutic potential of the UC-MSC secretome as a regenerative alternative to direct stem cell administration. This study aims to evaluate the safety and efficacy of the UC-MSC secretome in patients with severe ED who are unresponsive to sildenafil and to provide a more effective therapy option in the clinical management of ED.

## 2 Materials and methods

### 2.1 Ethical approval, study design, and clinical trial registration

The Institutional Review Board of Universitas Indonesia Hospital reviewed and approved this study protocol (protocol number 2025-06-120). All participants provided written informed consent at the time of enrollment. This study employed an experimental pre–posttest design, adapted from the methodology of von Schwarz et al., who evaluated stem cell injections in patients with ED (13).

### 2.2 Participants and eligibility criteria

A total of 15 subjects with severe ED unresponsive to sildenafil therapy were recruited using a consecutive sampling technique. Eligible participants were defined as follows:

1. Men aged 40–65 years. This age range was selected to enrich the cohort with patients with organic ED and established vascular risk factors, reduce heterogeneity related to psychogenic ED in younger men, and avoid the confounding effects of advanced age–related comorbidities and frailty that may independently affect penile hemodynamics and tissue stiffness.
2. Men with persistent severe ED with inadequate clinical response despite prior sildenafil therapy (100 mg taken on four separate occasions).
3. Those with organic ED with a baseline IIEF-5 score of 5–7.
4. Those with Type II DM with controlled glycemia (HbA1c < 7%).
5. Those with an IIEF-5 score <22 and not meeting the MCID threshold, defined as an IIEF-5 increase of <4 points.
6. Those with normal liver and renal function based on routine laboratory evaluations.

The exclusion criteria were as follows:

1. Penile anatomical abnormalities, such as Peyronie's disease, priapism, penile implants, or active skin irritation/wounds.
2. Active or significant systemic or local infections.
3. History of anticoagulant or antiplatelet use or other medications affecting coagulation.
4. History of systemic autoimmune disease or current immunosuppressive therapy.
5. Untreated hypogonadism or serum testosterone <200 ng/dL.
6. Uncontrolled hypertension or hypotension, defined as systolic blood pressure >170 or <90 mmHg, or diastolic blood pressure >100 or <50 mmHg.
7. Unstable cardiovascular disease, including myocardial infarction, unstable angina, or congestive heart failure, within the past 6 months.

### 2.3 Intervention: the UCMSC-derived secretome

The intervention used in this study was a UCMSC-derived secretome (UCMSC–Secretome; PT Kalbe Stem Cell & Biotechnology), provided as a 1.5-mL sterile injectable solution based on its Certificate of Analysis (DPP4-01-GF-LUCM.00). The product comprises conditioned medium containing extracellular bioactive molecules secreted by UC-MSCs, including growth factors, cytokines, chemokines, soluble proteins, lipids, and extracellular vesicles. Quantitatively confirmed components included bFGF (89–144 pg/mL), TGF-β1 (438–577 pg/mL), KGF (70–75 pg/mL), HGF (1,152–1,290 pg/mL), and procollagen (467–512 ng/mL). Additional bioactive molecules such as adiponectin, angiopoietins, BMP-4, cathepsins, complement factors, DPPIV, EN-RAGE, IL-1β, IL-6, IL-10, IGFBPs, CXCL8/IL-8, and other immunomodulatory and regenerative mediators that support the product's proangiogenic, anti-inflammatory, and tissue-repair biological activity were qualitatively identified.

### 2.4 Procedure and data collection

All clinical and ultrasonographic assessments were performed at standardized time points: 1 day before the procedure (D–1), the day of injection before the procedure (D0), 1 day after the procedure (D + 1), and 1 month following the intracavernosal intervention (D + 30). Penile hemodynamics [peak systolic velocity [PSV], resistive index [RI], and cavernosal artery diameter], intima-media thickness (IMT) measurements, and elastography parameters [shear wave elastography [SWE] and strain ratio [SR]] were assessed at D0 and D + 30.

The enrolled patients received a single intracavernosal injection of the UC-MSC secretome. A volume of 0.5 mL of the secretome solution was injected into each corpus cavernosum using a 27-gauge needle under sterile conditions, performed by a urologist experienced in intracavernosal procedures.

The safety parameters monitored in this study were pain, hematoma, local bleeding, and infection. The IIEF-5 questionnaire, EHS, morning erection frequency, SPL, FPL, and penile circumference were used to assess efficacy outcomes. Penile Doppler Color Ultrasound (PDCU) examinations were conducted using a high-resolution ultrasound device equipped with a 7–12 MHz linear transducer. All PDCU assessments were performed in a temperature-controlled room in the flaccid state after 10 min of rest. Spectral Doppler evaluation included measurements of PSV and EDV in the bilateral cavernosal arteries. RI was calculated using the standard formula [RI = (PSV−EDV)/PSV]; when EDV was absent, RI was recorded as 1 in accordance with Doppler conventions.

Cavernosal artery diameter and IMT were measured at the proximal, mid, and distal segments of both corpora. Elastography assessments included SWE and SR measurements performed at the same anatomical levels. Each elastography parameter was measured thrice, and the mean value was used to minimize intraobserver variability.

### 2.5 Estimation of sample size

In this pilot pre–post interventional study, the sample size was estimated using the standard formula for determining the minimum required sample in studies comparing two means:

$$n={\left[\frac{(Z_{\alpha }+Z_{\beta })\times S}{\Delta }\right]}^{2}$$

where *Z_α_* represents the significance level (*α* = 0.05), Z*_β_* denotes the desired statistical power (80%), *S* is the standard deviation of the outcome variable, and *Δ* is the expected mean difference to be detected. Based on research by Yiou et al., it was reported clinically meaningful changes in IIEF-5 of approximately 4 points and a standard deviation of 4, according to previous studies (17). Thus, the minimum calculated sample size was 10 (17). The preliminary exploration for this single-arm study will start with 10 participants.

### 2.6 Statistical analysis

The data were analyzed using descriptive analysis and were presented as mean ± SD for normal distribution variables and median (min-max) for non-normally distributed variables according to the Shapiro–Wilk normality test. The paired t-test was used to compare variables with a normal distribution, whereas non-normally distributed data were analyzed using the Wilcoxon signed-rank test. A *p*-value <0.05 was considered statistically significant. Statistical analyses were performed using the Statistical Package for the Social Sciences version 22.

## 3 Results

This study involved 15 male patients with severe ED who were non-responsive to sildenafil, with a mean age of 60.60 ± 3.50 years. Table 1 summarizes the baseline patient characteristics.

**Table 1:** Characteristics of the study participants.

Characteristics	*N* = 15
Age (mean ± SD)	62.50 ± 3.50
AST [median (min–max)]	21.5 (14–35)
ALT [median (min–max)]	20 (9–49)
Urea (mean ± SD)	18.22 ± 4.87
Creatinine (mean ± SD)	1.07 ± 0.09
Fasting plasma glucose (mean ± SD)	121.20 ± 11.30
HbA1c (mean ± SD)	6.15 ± 0.62
Prostate-specific antigen [median (min–max)]	1.72 (1.22–1.92)
Testosterone (mean ± SD)	608.42 ± 281.62

### 3.1 Safety and efficacy (sign, symptoms, and questionnaire results)

One study aimed to measure the effect of the administration of the UC-MSC secretome on outcomes such as pain, hematoma, local bleeding, infection, and changes in IIEF-5, EHS, and morning erections. The Wilcoxon analysis was used to compare outcomes before and after the intervention at various times (D−1, D0, D + 1, and D + 30) (Table 2).

**Table 2:** Results of the Wilcoxon test before and after the administration of the mesenchymal stem cell secretome.

Outcomes	The umbilical cord mesenchymal stem cell secretome				*p*-Value					
	D−1 (A)	D0 (B)	D + 1 (C)	D + 30 (D)	A−B	A−C	A−D	B−C	B−D	C−D
Local bleeding										
No bleeding	15	15	15	15	1.000	1.000	1.000	1.000	1.000	1.000
Leakage	0	0	0	0						
Active bleeding	0	0	0	0						
Hematoma										
<1 mm^2^	15	15	15	15	1.000	1.000	1.000	1.000	1.000	1.000
1 mm^2^	0	0	0	0						
>1 or <2 mm^2^	0	0	0	0						
>2 mm^2^	0	0	0	0						
Pain										
No pain	15	0	15	15	0.002	1.000	1.000	0.002	0.002	1.000
Mild	0	15	0	0						
Moderate	0	0	0	0						
Severe	0	0	0	0						
Infection										
No swelling	15	15	15	15	1.000	1.000	1.000	1.000	1.000	1.000
1 sign of infection	0	0	0	0						
2 Signs of infection	0	0	0	0						
3 Signs of infection	0	0	0	0						
IIEF-5										
1–4	0	0	0	0	1.000	1.000	0.005	0.180	0.004	0.003
5	8	8	6	0						
6	2	2	3	0						
7	5	5	6	0						
8	0	0	0	0						
9	0	0	0	5						
10	0	0	0	2						
11	0	0	0	0						
12	0	0	0	2						
13	0	0	0	0						
14	0	0	0	2						
15	0	0	0	2						
16	0	0	0	2						
17–25	0	0	0	0						
EHS										
Slightly enlarged	15	15	15	0	1.000	1.000	0.004	1.000	0.004	0.004
Not strong enough for penetration	0	0	0	10						
Hard enough for penetration	0	0	0	5						
Rigid	0	0	0	0						
Morning erection										
None	15	15	3	0	1.000	0.004	0.003	0.004	0.003	0.065
Present	0	0	12	15						

The administration of umbilical cord–derived mesenchymal stem cell secretome showed significant improvements in several parameters in patients with severe ED unresponsive to sildenafil. There were no changes in local bleeding, hematoma, or infection outcomes (*p* = 1.000); thus, further analysis could not be performed. However, there were significant differences in pain between D−1 and D0, D0 and D + 1, and D0 and D + 30 (*p* = 0.002). The EHS also increased significantly between D−1 and D + 30, D0 and D + 30, and D + 1 and D + 30 (*p* = 0.004). Similar improvements were observed in the IIEF-5 scores and the occurrence of morning erections up to D + 30 (*p* = 0.003 for IIEF-5 and *p* = 0.003–0.004 for morning erections). These findings indicate that secretome therapy can alleviate pain, increase erection hardness, improve erectile function, and increase morning erection occurrence in patients with severe ED who are unresponsive to sildenafil.

### 3.2 Physical examination: stretched penis length, flaccid penis length, and penis circumference

This study aimed to measure the effect of the administration of the UC-MSC secretome on SPL, FPL, and penis circumference. The paired t-test was used to compare the outcomes before and after the intervention at various time points (Table 3).

**Table 3:** Paired *t*-test of SPL, FPL, and penis circumference before and after secretome injection.

SPL	Mean	SD	Delta	*P*
Preinjection SPL (cm)	11,071	1,742	0,715	am
Postinjection SPL (cm)	11,786	1,729		
FPL	Mean	SD	Delta	*P*
Preinjection FPL (cm)	7,571	1,272	0,643	0,022
Postinjection FPL (cm)	8,214	0,859		
Penis circumference	Median	Min–max	Delta	*P*
Preinjection penis circumference (cm)	8,0	8,0–9,0	0,64	0,014
Postinjection penis circumference (cm)	8,5	8,5–10,0		

The results showed significant differences in SPL, FPL, and penis circumference among patients with ED who did not respond to PDE5i before and after the administration of the UC-MSC secretome.

### 3.3 USG-based assessment

#### 3.3.1 Peak systolic velocity (PSV) and diameter of the cavernosum artery

**Figure 1:**
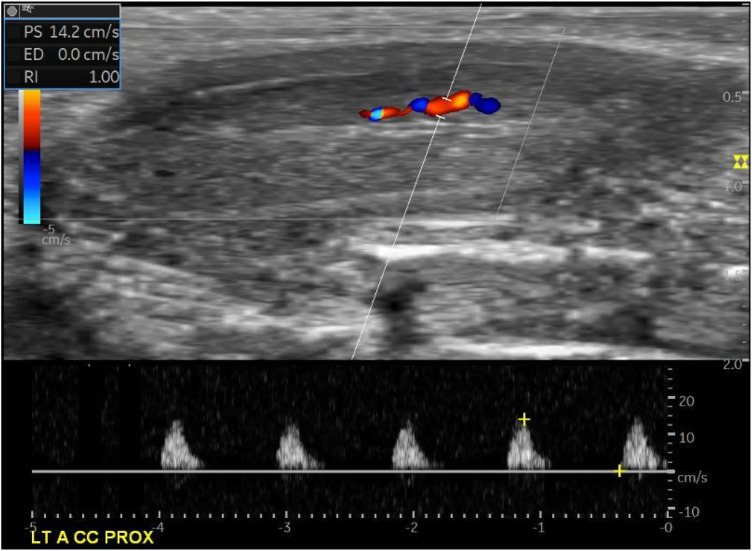
A monophasic spectral waveform was observed in a patient with cavernosal artery erectile dysfunction in the flaccid state.

The study subjects underwent a PCDU examination while in the flaccid state to assess both research parameters, namely, PSV and diameter of the proximal side cavernosum artery lumen, before and 1 month after the intracavernosal injection intervention. The difference in the PSV of the right and left cavernosum arteries was analyzed using the paired t-test because the results of the data normality test were based on the Shapiro–Wilk test, which showed a normal data distribution (*p* > 0.05). The difference in the diameter of the right cavernosum artery lumen on the proximal side was analyzed using the paired t-test because the results of the data normality test that were based on the Shapiro–Wilk test showed a normal data distribution (*p* > 0.05). Although the left cavernosum artery lumen diameter on the proximal side had an abnormal data distribution, it was analyzed using the Wilcoxon test (Figure 1).

The diameter of the cavernosum artery in patients with severe ED non-responsive to sildenafil before and after the administration of the UC-MSC secretome increased slightly, that is, 0.006 cm for the right and 0.003 cm for the left. The test results showed no statistically significant difference in the cavernosum artery diameter (*p* > 0.05). (Table 4).

**Table 4:** The paired *t*-test of cavernosal artery PSV and cavernosal artery diameter in patients before and after secretome injection.

PSV	Mean	SD	difference	*P*
Preinjection of PSV (right)	9.157	2.379	2.27	0.005
Postinjection PSV (right)	11.428	2.167		
Preinjection of PSV (left)	9.100	3.079	3.01	0.001
Postinjection PSV (left)	12.114	3.629		
Cavernosum artery diameter	Mean	SD	difference	*P*
Preinjection right lumbar	0.048	0.014	0.006	0.386
Postinjection right lumen	0.054	0.009		
Preinjection left lumbar	0.051	0.003	0.003	0.157
Postinjection left lumen	0.054	0.007		

#### 3.3.2 Resistive index (RI) and intima-media thickness (IMT)

The research subjects underwent a penile ultrasound examination to assess the two research parameters in the form of RI, with the formula RI = (PSV − EDV)/PSV and IMT of the right and left proximal-mid-distal cavernosal arteries before and 1 month after intracavernosal secretome injection.

The RI value could not be analyzed because the EDV value did not appear in the examination. Therefore, the RI was recorded as 1, but the PSV value changed before and after the intracavernosal secretome injection.

The difference in the PSV of the right proximal-mid-distal and left proximal-mid cavernosal arteries was analyzed using the paired t-test because the results of the data normality test based on the Shapiro–Wilk test showed normal data distribution results (*p* > 0.05). Meanwhile, the PSV of the left distal cavernosal artery had an abnormal data distribution; therefore, it was analyzed using the Wilcoxon test.

The difference in the IMT of the right proximal-mid and left distal cavernosum arteries was analyzed using the paired t-test because the results of the data normality test based on the Shapiro–Wilk test showed normal data distribution results (*p* > 0.05). Although the IMT of the right distal and left proximal-mid cavernosum arteries had non-normal data distributions, they were analyzed using the Wilcoxon test (Table 5).

**Table 5:** Paired *t*-test of cavernosal artery PSV in patients with severe ED nonresponsive to sildenafil before and after UC-MSC secretome administration.

PSV	Mean	SD	Difference	*P*
PrepsvRP	9,16	2,38	2,27	0,005
PostpsvRP	11,43	2,17		
PrepsvRM	6,76	2,16	0,68	0,051
PostpsvRM	7,44	1,91		
PrepsvRD	4,94	1,49	0,95	0,011
PostpsvRD	5,89	1,04		
PrepsvLP	9,10	3,08	3,01	0,001
PostpsvLP	12,11	3,63		
PrepsvLM	7,13	2,37	2,43	0,038
PostpsvLM	9,56	3,86		
PrepsvLD	5,51	0,95	1,00	0,046
PostpsvLD	6,51	2,10		

#### 3.3.3 SWE and strain ratio

**Figure 2:**
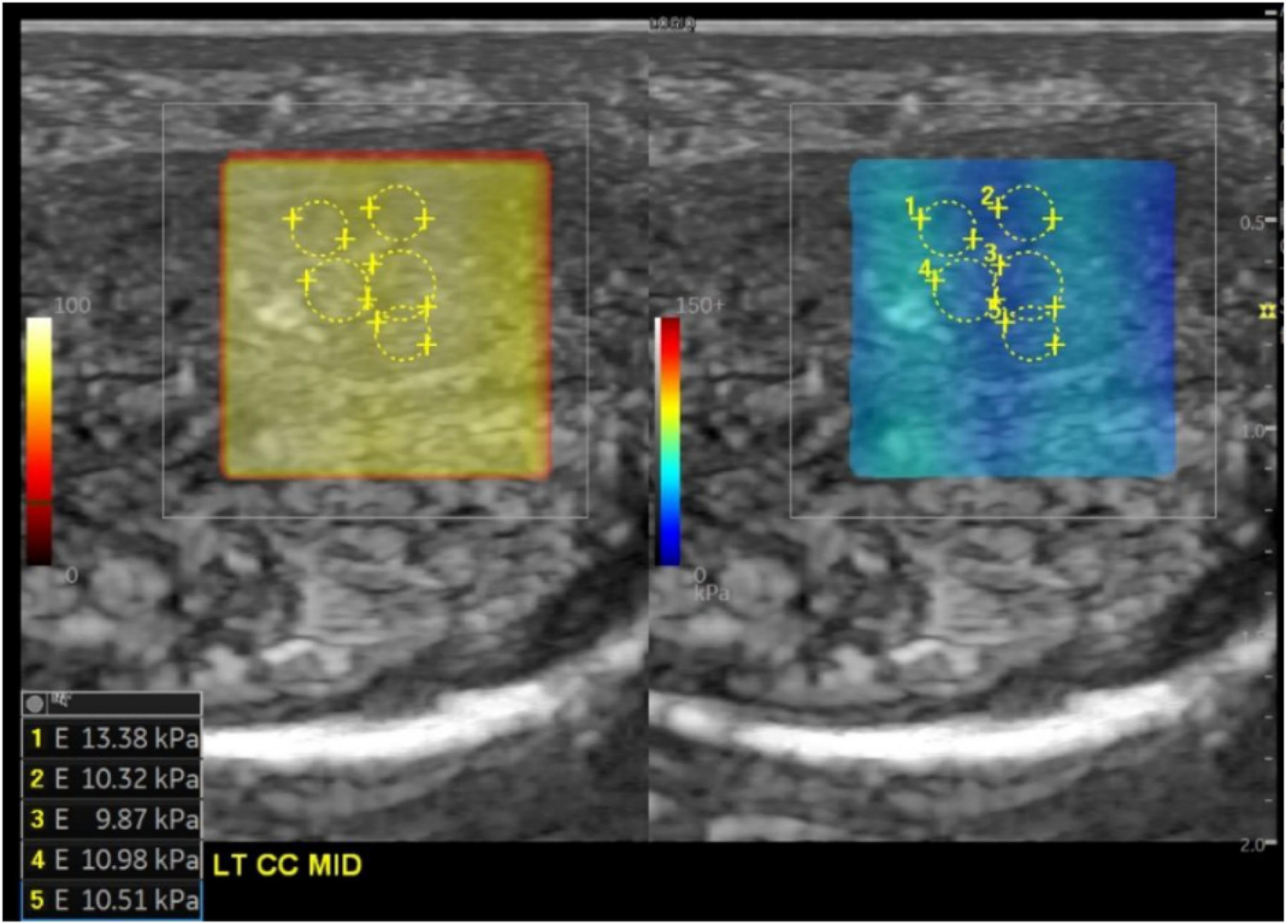
SWE measurements from the left corpus cavernosum showed multiple examples of regions of interest (yellow lines) and color map elasticity in the square region.

The SWE results showed a normal data distribution (*p* > 0.05) on the right side and an abnormal distribution (*p* < 0.05) in the left mid-distal SWE data. The right and left proximal SWE data were subjected to the Wilcoxon test when the data distribution was not normal and to the paired t-test when the data distribution was normal. The results revealed a statistically significant decrease in SWE values in both the right and left corpus cavernosum, with *p*-values of 0.018 and 0.022 (*p*-value <0.05) (Table 6, Figure 2).

**Table 6:** SWE results before and after secretome injection.

Parameter US	Preinjection		Postinjections		*P*-value
	Mean ± SD	Median (min–max)	Mean ± SD	Median (min–max)	
SWE right proximal	9.25 ± 1.55 kPa	9.01 (7.30–11.50) kPa	8.66 ± 1.44 kPa	8.90 (6.86–10.60) kPa	0.018
SWE right midway	9.84 ± 2.10 kPa	8.91 (7.80–13.20) kPa	8.78 ± 2.21 kPa	7.67 (6.57–12.40) kPa	0.009
SWE right distal	10.31 ± 3.61 kPa	9.50 (6.30–15.50) kPa	8.53 ± 2.08 kPa	9.27 (5.70–11.20) kPa	0.034
Left proximal SWE	10.56 ± 2.22 kPa	10.50 (8.30–14.10) kPa	8.96 ± 1.50 kPa	8.26 (7.57–11.70) kPa	0.018
SWE left midway	10.72 ± 1.63 kPa	11.01 (8.89–13.07) kPa	9.24 ± 1.08 kPa	8.99 (8.11–11.01) kPa	0.020
SWE left distal	11.09 ± 2.80 kPa	12.80 (6.20–13.60) kPa	9.64 ± 2.05 kPa	9.40 (6.10–12.20) kPa	0.039

**Figure 3:**
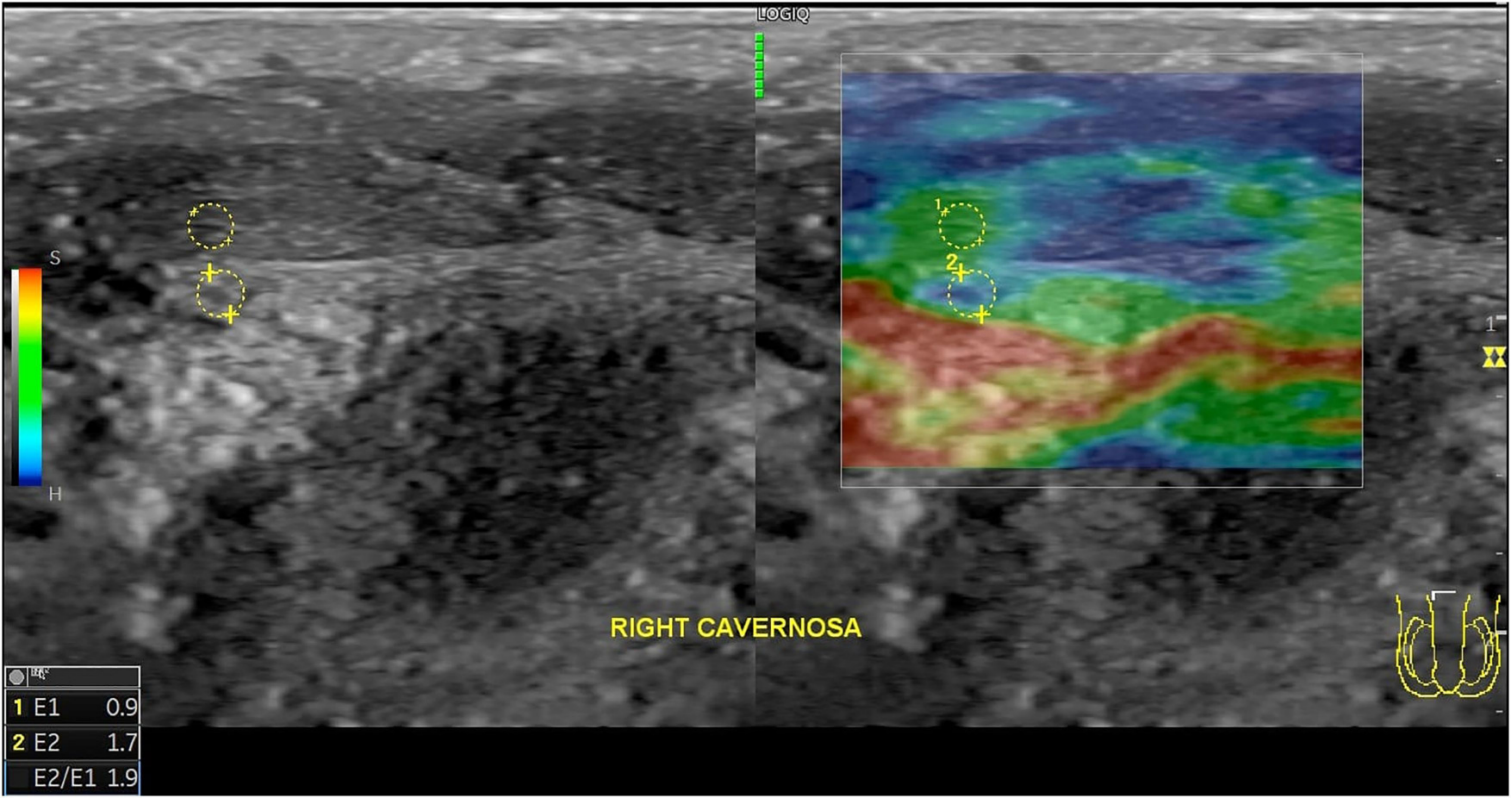
Strain ratio measurement images on the left corpus cavernosum in the region of interest examples (yellow lines) and color map elasticity in the square region.

Table 7 shows that the difference in SR was analyzed using the Shapiro–Wilk normality test, which showed a normal data distribution (*p* > 0.05) in the left SR and an abnormal data distribution (*p* < 0.05) in the right SR. Subsequently, a paired t-test and a Wilcoxon test were conducted on the left and right SR, respectively. The results indicated a semiquantitative decrease in stiffness values in both the right and left SR corpus, with *p*-values of 0.018 and 0.022, respectively, indicating significant differences (*p*-value <0.05) (Figure 3).

**Table 7:** Results of strain ratio before and after secretome injection.

Parameter US	Preinjection		Postinjection		*P*-value
	Mean ± SD	Median (min–max)	Mean ± SD	Median (min–max)	
Right strain ratio	1.53 ± 0.46	1.50 (1.00–2.30)	1.13 ± 0.28	1.10 (0.80–1.70)	0.018
Left strain ratio	1.69 ± 0.39	1.60 (1.20–2.30)	1.30 ± 0.30	1.20 (1.00–1.80)	0.022

## 4 Discussion

This study included subjects with severe ED who were unresponsive to sildenafil, with a mean age of 60.60 years, placing them in the older adult category. In this age group, declines in hormonal levels and physiological function—including erectile capacity—are commonly observed. Laboratory examinations showed that liver function remained within normal limits, which is essential for ensuring efficient drug metabolism and preventing drug accumulation-related toxicity (19). Kidney function was also normal, with mean urea and creatinine values of 18.22 and 1.07, respectively, supporting adequate secretome metabolite excretion. A previous study suggested that preserved kidney function helps prevent the buildup of potentially toxic metabolites (20).

The therapy utilized MSC secretome administered directly into the erectile tissue of the corpora cavernosa, enabling maximal interaction with the target site without prior hepatic metabolism. Kumar et al. reported that direct intracavernosal injection of MSC-derived secretome allowed bioactive components, such as cytokines and growth factors, to immediately interact with local smooth muscle and endothelial cells, thereby enhancing vascularization and erectile function through regenerative mechanisms (21). Some components may enter the systemic circulation, where the hepatic and renal systems further influence distribution and metabolism. Impaired liver or kidney function may therefore reduce the effectiveness and distribution of the therapy (22).

Therapy safety was assessed using pain, local bleeding, hematoma, and infection as parameters. The Wilcoxon test results showed no significant differences in hematoma, local bleeding, and infection between D−1, D0, D + 1, and D + 30 (*p* > 0.05), indicating that secretome injection is safe and does not induce complications within 1 month postprocedure. A previous study also reported that the MSC secretome can be administered safely without patients experiencing significant adverse effects (11). Pain scores showed a mild increase on D0 immediately after injection, followed by a reduction the next day, indicating that pain was only transient.

Significant improvements in both the International Index of Erectile Function (IIEF-5) and the EHS after 30 days demonstrated therapeutic effectiveness. The IIEF-5 score increased from 5.7 (severe ED) to 11.3 (moderate ED) at D + 30, which is consistent with the findings of Schwarz et al., who reported improved erectile function following MSC secretome therapy (13). Similarly, improvements in the EHS—from score 1 (slightly enlarged) to scores 2–3 at D + 30—align with the findings of Cheng et al., which associated increases in the EHS with growth factors, such as VEGF and FGF, that promote angiogenesis and cavernous tissue vascularization (23).

This study also found significant increases in SPL, FPL, and penile circumference in patients with ED unresponsive to PDE5 inhibitors following the administration of the umbilical cord–derived MSC secretome. The SPL increased by 0.715 cm, FPL by 0.643 cm, and penile circumference by 0.50 cm on D + 30. These improvements were influenced by both regenerative and paracrine factors. SPL changes were more related to paracrine-mediated alterations in tissue elasticity, whereas increases in FPL and penile circumference were dominated by regenerative mechanisms that enhanced cellular proliferation in the corporeal and glanular tissues. According to Von Schwarz, stem cell therapy increases VEGF concentrations, leading to endothelial cell proliferation and enhanced endothelial content in cavernous tissue—an indicator of vascular regeneration. Stem cell therapy is generally associated with the secretion of numerous growth factors that support tissue repair, and it also modulates immune responses by reducing inflammatory mediators (e.g., IL-6, IL-1β, TNF-α) (15).

The flaccid-state PSV was used as a screening tool for ED. A study by Chen et al. aimed to determine the potential value of sonographic measurements of a flaccid penis in diagnosing arteriogenic ED in an Asian population (24). A Doppler analysis of the flaccid penis is relatively inexpensive, takes less time than a dynamic examination, and is free from side effects such as priapism. This study evaluated the effect of the UC-MSC secretome on the PSV of the right and left proximal cavernous arteries in the flaccid state. Before intervention, the mean PSV was 9.157 cm/s in the right artery and 9.100 cm/s in the left artery, both below the threshold predicting reduced dynamic PSV. A statistical analysis showed significant improvements in the flaccid PSV of both the right (*p* = 0.005) and left (*p* = 0.001) cavernous arteries at 1 month post-treatment.

These findings align with those of clinical trials by Levy et al., who used an intracavernosal injection of placental matrix-derived MSCs (PM-MSCs) in patients with ED who were intolerant to oral therapy and unwilling to undergo penile prosthesis implantation. The initial dynamic PSV values (after trimix injection) ranged from 23.1 to 49.3 cm/s. At week 6, PSV increased to 25.5–56.5 cm/s, and at 3 months, it rose to 32.5–66.7 cm/s (*p* < 0.05). By month 6, the values had further increased to 50.7–73.9 cm/s (*p* < 0.01) (25). Similarly, Al Demour et al. demonstrated that two intracavernosal injections of allogeneic Wharton's Jelly MSCs at 30-day intervals significantly increased flaccid PSV from 12.06 to 16.35 cm/s at 3 months (*p* = 0.0332) (26).

Color Doppler ultrasonography remains the primary method for assessing arterial flow to the corpora cavernosa; however, the evaluation of tunica IMT may provide earlier detection of vascular pathology in ED, even when PSV is still normal (27, 28). Research by Caretta et al. showed that cavernous artery morphology evaluation can detect atherosclerotic plaques and assess variations in lumen diameter following pharmacologic stimulation. A cavernous IMT ≥0.3 mm predicts plaque presence with 100% sensitivity and 76.4% specificity—superior to PSV—and IMT correlates negatively with PSV (28).

The corpus cavernosum contains smooth muscle cells (SMCs) and collagen, with SMCs playing a critical functional role. Aging and disease reduce SMC number and degrade elastic and collagen fibers, contributing to sinusoidal obstruction, fibrosis, and impaired smooth muscle relaxation. This hinders blood filling in the cavernous sinusoids. These structural changes alter penile tissue stiffness, which is measurable through elastography. Greater SMC density results in more elastic and less stiff tissue. SWE provides real-time assessment of tissue stiffness by measuring shear wave propagation velocities (17, 18). Zhang et al. demonstrated that reduced SMC content led to increased SWE measurements. In this study, secretome administration improved penile tissue elasticity through regenerative and paracrine mechanisms, increasing SMC content and reducing elastography values (strain ratio and kPa). Previous studies have shown that the secretome possesses angiogenic, developmental, antifibrotic, immunomodulatory, antiapoptotic, and anti-inflammatory properties, enabling adaptive remodeling of damaged tissue structures (9).

Intracavernosal administration of umbilical cord mesenchymal stem cell (UCMSC)–derived secretome represents a biologically plausible regenerative approach that may target the underlying structural and molecular derangements of erectile dysfunction (ED), rather than providing only transient hemodynamic support as observed with phosphodiesterase-5 inhibitors (PDE5i). Although PDE5i enhance nitric oxide–mediated vasodilation, they do not reverse endothelial dysfunction, smooth muscle depletion, or cavernosal fibrosis, which are central features of organic ED. In contrast, the UC-MSC secretome contains bioactive growth factors and extracellular vesicles that have been shown to promote angiogenesis, modulate inflammation, reduce apoptosis, and support smooth muscle restoration in preclinical models (29). Nevertheless, given the limited sample sizes, heterogeneity of protocols, and short follow-up durations in existing studies, the findings in this study should be interpreted with caution. Well-designed, adequately powered randomized controlled trials with standardized outcome measures are required to determine the long-term efficacy, safety, and clinical positioning of UCMSC secretome therapy within the current treatment algorithm for ED.

## Data Availability

The raw data supporting the conclusions of this article will be made available by the authors, without undue reservation.

## References

[1] Al-Shaiji TF. Breaking the ice of erectile dysfunction taboo: a focus on clinician-patient communication. J Patient Exp. (2022) 9:23743735221077512. 10.1177/23743735221077512 35128040 PMC8808006

[2] Wang CM Wu BR Xiang P Xiao J Hu XC. Management of male erectile dysfunction: from the past to the future. Front Endocrinol (Lausanne). (2023) 14:1148834. 10.3389/fendo.2023.1148834 36923224 PMC10008940

[3] Muneer A Kalsi J Nazareth I Arya M. Erectile dysfunction. Br Med J. (2014) 348:g129. 10.1136/bmj.g129 24468580

[4] Birowo P Deswanto IA Rasyid N. Epidemiology of erectile dysfunction: a cross-sectional web-based survey conducted in an Indonesian national referral hospital. F1000Res. (2019) 8:817. 10.12688/f1000research.19031.1

[5] Kessler A Sollie S Challacombe B Briggs K Van Hemelrijck M. The global prevalence of erectile dysfunction: a review. BJU Int. (2019) 124(4):587–99. 10.1111/bju.14813 31267639

[6] Li JZ Maguire TA Zou KH Lee LJ Donde SS Taylor DG. Prevalence, comorbidities, and risk factors of erectile dysfunction: results from a prospective real-world study in the United Kingdom. Int J Clin Pract. (2022) 2022:5229702. 10.1155/2022/5229702 35693549 PMC9159135

[7] Burnett AL Nehra A Breau RH Culkin DJ Faraday MM Hakim LS Erectile dysfunction: AUA guideline. J Urol. (2018) 200(3):633–41. 10.1016/j.juro.2018.05.004 29746858

[8] Sun DZ Abelson B Babbar P Damaser MS. Harnessing the mesenchymal stem cell secretome for regenerative urology. Nat Rev Urol. (2019) 16(6):363–75. 10.1038/s41585-019-0169-3 30923338 PMC7027199

[9] Mukti AI Ilyas S Warli SM Putra A Rasyid N Munir D Mesenchymal stem cells enhance vascular endothelial growth factor-A, endothelial nitric oxide synthase, and HSP70 expression in improving erectile dysfunction in streptozotocin-induced diabetic rats. Open Access Maced J Med Sci. (2019) 9:1174–80. 10.3889/oamjms.2021.7801

[10] Krawczenko A Klimczak A. Adipose tissue-derived mesenchymal stem/stromal cells and their contribution to angiogenic processes in 71 tissue regeneration. Int J Mol Sci. (2022) 23:5. 10.3390/ijms23052425 PMC8910401 35269568

[11] Margiana R Abdullah MF Pakpahan C. Analyzing the current umbilical cord-derived mesenchymal stem cell secretome evidence for erectile dysfunction management. Biomol Health Sci J. (2023) 6:141–6. 10.4103/bhsj.bhsj_10_23

[12] Gordon CM Carey MP. Penile tumescence monitoring during morning naps to assess male erectile functioning: an initial study of healthy men of varied ages. Arch Sex Behav. (1995) 24(3):291–307. 10.1007/BF01541601 7611847

[13] Utomo E Blok BF Pastoor H Bangma CH Korfage IJ. The measurement properties of the five-item International Index of Erectile Function (IIEF-5): a Dutch validation study. Andrology. (2015) 3(6):1154–9. 10.1111/andr.12112 26453539

[14] Varela CG Mateos Yeguas LA Rodríguez IC Durán Vila MD. Penile Doppler ultrasound for erectile dysfunction: technique and interpretation. Am J Roentgenol. (2020) 214(5):1112–21. 10.2214/AJR.19.22141 31990215

[15] Von Schwarz ER Busse N Angelus KM Omair A Von Schwarz AA Bogaardt PC. Intracavernous injection of stem cell-derived bioactive molecules for erectile dysfunction—a pilot phase nonrandomized controlled trial. J Men’s Health. (2021) 17(4):99–108. 10.31083/jomh.2021.090

[16] Prezioso D Iacono F Russo U Romeo G Ruffo A Russo N Evaluation of penile cavernosal artery intima-media thickness in patients with erectile dysfunction. A new parameter in the diagnosis of vascular erectile dysfunction our experience on 59 cases. Archivio Italiano di Urologia e Andrologia. (2014) 86:9–14. 10.4081/aiua.2014.1.9 24704924

[17] Yiou R Hamidou L Birebent B Bitari D Lecorvoisier P Contremoulins I Safety of intracavernous bone marrow mononuclear cells for postradical prostatectomy erectile dysfunction: an open dose-escalation pilot study. Eur Urol. (2016) 69(6):988–91. 10.1016/j.eururo.2015.09.026 26439886

[18] Rosen RC Allen KR Ni X Araujo AB. Minimal clinically important differences in the erectile function domain of the International Index of Erectile Function scale. Eur Urol. (2011) 60(5):1010–6. 10.1016/j.eururo.2011.07.053 21855209

[19] Zheng L Gong H Zhang J Guo L Zhai Z Xia S Strategies to improve the therapeutic efficacy of mesenchymal stem cell-derived extracellular vesicle (MSC-EV): a promising cell-free therapy for liver disease. Front Bioeng Biotechnol. (2023) 11:1322514. 10.3389/fbioe.2023.1322514 38155924 PMC10753838

[20] Beer L Mildner M Ankersmit HJ. Cell secretome-based drug substances in regenerative medicine: when regulatory affairs meet basic science. Ann Transl Med. (2017) 5(7):170. 10.21037/atm.2017.03.50 28480206 PMC5401661

[21] Kumar LP Kandoi S Misra R Vijayalakshmi S Rajagopal K Verma RS. The mesenchymal stem cell secretome: a new paradigm toward a cell-free therapeutic mode in regenerative medicine. Cytokine Growth Factor Rev. (2019) 46(4):1–9. 10.1016/j.cytogfr.2019.04.002 30954374

[22] Trzyna A Banaś-Ząbczyk A. Adipose-derived stem cells secretome and its potential application in “stem cell-free therapy.” Biomolecules. (2021) 11(6):878. 10.3390/biom11060878 34199330 PMC8231996

[23] Cheng H Niu Z Xin F Yang L Ruan L. A new method to quantify penile erection hardness: real-time ultrasonic shear wave elastography. Transl Androl Urol. (2020) 9(4):1735–42. 10.21037/tau-20-1096 32944534 PMC7475665

[24] Chen LD Pan FS Zhou LY Liu YB Lv JY Xu M Value of flaccid penile ultrasound in screening for arteriogenic impotence: a preliminary prospective study. BMC Med Imaging. (2018) 18(1):1–7. 10.1186/s12880-018-0284-2 30400881 PMC6219149

[25] Levy JA Marchand M Iorio L Zribi G Zahalsky MP. Effects of stem cell treatment in human patients with peyronie disease. J Osteopath Med. (2015) 115:e8–13. 10.7556/jaoa.2015.124 26414724

[26] Al Demour S Adwan S Jafar H Rahmeh R Alhawari H Awidi A. Safety and efficacy of intracavernous injections of allogeneic Wharton’s Jelly-derived mesenchymal stem cells in diabetic patients with erectile dysfunction: phase 1/2. Clinical Trial Urol Int. (2021) 105:935–43. 10.1159/000517364 34384079

[27] Foresta C De Toni L Biagioli A Ganz F Magagna S Caretta N. Increased levels of osteocalcin-positive endothelial progenitor cells in patients affected by erectile dysfunction and cavernous atherosclerosis. J Sex Med. (2010) 7:751–7. 10.1111/j.1743-6109.2009.01520.x 19796016

[28] Caretta N Palego P Schipilliti M Ferlin A Di Mambro A Foresta C. Cavernous artery intima-media thickness: a new parameter in the diagnosis of vascular erectile dysfunction. J Sex Med. (2009) 6(4):1117–26. 10.1111/j.1743-6109.2008.01112.x 19067788

[29] Margiana R Pilehvar Y Amalia FL Lestari SW Supardi S I'tishom R. Mesenchymal stem cell secretome: a promising therapeutic strategy for erectile dysfunction? Asian J Urol. (2024) 11(3):391–405. 10.1016/j.ajur.2024.02.003 39139521 PMC11318444

